# The Arabidopsis transcription factor AINTEGUMENTA orchestrates patterning genes and auxin signaling in the establishment of floral growth and form

**DOI:** 10.1111/tpj.14769

**Published:** 2020-05-05

**Authors:** Beth A. Krizek, Ivory C. Blakley, Yen‐Yi Ho, Nowlan Freese, Ann E. Loraine

**Affiliations:** ^1^ Department of Biological Sciences University of South Carolina Columbia SC 29208 USA; ^2^ Department of Bioinformatics and Genomics University of North Carolina at Charlotte Charlotte NC 28223 USA; ^3^ Department of Statistics University of South Carolina Columbia SC 29208 USA

**Keywords:** *Arabidopsis thaliana*, flower development, AINTEGUMENTA (ANT), AINTEGUMENTA‐LIKE (AIL)/PLETHORA (PLT), RNA‐Seq, ChIP‐Seq, organ polarity, organ growth, auxin signaling

## Abstract

Understanding how flowers form is an important problem in plant biology, as human food supply depends on flower and seed production. Flower development also provides an excellent model for understanding how cell division, expansion and differentiation are coordinated during organogenesis. In the model plant *Arabidopsis thaliana*, floral organogenesis requires AINTEGUMENTA (ANT) and AINTEGUMENTA‐LIKE 6 (AIL6)/PLETHORA 3 (PLT3), two members of the Arabidopsis AINTEGUMENTA‐LIKE/PLETHORA (AIL/PLT) transcription factor family. Together, ANT and AIL6/PLT3 regulate aspects of floral organogenesis, including floral organ initiation, growth, identity specification and patterning. Previously, we used RNA‐Seq to identify thousands of genes with disrupted expression in *ant ail6* mutant flowers, indicating that ANT and AIL6/PLT3 influence a vast transcriptional network. The immediate downstream targets of ANT and AIL6/PLT3 in flowers are unknown, however. To identify direct targets of ANT regulation, we performed an RNA‐Seq time‐course experiment in which we induced ANT activity in transgenic plants bearing an ANT‐glucocorticoid receptor fusion construct. In addition, we performed a ChIP‐Seq experiment that identified ANT binding sites in developing flowers. These experiments identified 200 potential ANT target genes based on their proximity to ANT binding sites and differential expression in response to ANT. These 200 candidate target genes were involved in functions such as polarity specification, floral organ development, meristem development and auxin signaling. In addition, we identified several genes associated with lateral organ growth that may mediate the role of ANT in organ size control. These results reveal new features of the ANT transcriptional network by linking ANT to previously unknown regulatory targets.

## Significance Statement

Flower development has been extensively studied, but our knowledge of gene regulatory networks generating floral organs with characteristic forms remains incomplete. This study used a highly targeted genomic approach to identify 200 genes likely to be direct targets of regulation by AINTEGUMENTA (ANT), a transcription factor regulating flower patterning and growth. Our set of identified target genes include those with known roles in polarity specification, floral organ development, lateral organ growth and auxin signaling.

## INTRODUCTION

Flowers supply fruits, seeds and grains to the human diet, and are a subject of fascination for their beauty and morphological diversity. Molecular genetic studies in Arabidopsis have identified many regulatory factors that control the initiation and subsequent development of flowers. Flower primordia arise in the periphery of the inflorescence meristem at sites of auxin maxima, during the reproductive phase of the plant life cycle (Benkova *et al.*, 2003; Reinhardt *et al.*, 2003; Heisler *et al.*, 2005). At these sites, AUXIN RESPONSE FACTOR 5/MONOPTEROS (ARF5/MP) upregulates the expression of *LEAFY* (*LFY)* and two *AINTEGUMENTA‐LIKE/PLETHORA* (*AIL/PLT*) genes, *AINTEGUMENTA* (*ANT*) and *AINTEGUMENTA‐LIKE 6* (*AIL6*)/*PLETHORA 3* (*PLT3*), to specify these primordia as flowers and promote their outgrowth (Yamaguch *et al.*, 2013). Within flower primordia, floral organ primordia are initiated at defined positions within whorls and subsequently adopt one of four fates according to the ABCE model (reviewed by Krizek and Fletcher, 2005). Class‐A and ‐E gene activities in whorl 1 specify sepal identity, class‐A, ‐B and ‐E gene activities in whorl 2 specify petal identity, class‐B, ‐C and ‐E gene activities in whorl 3 specify stamen identity, and class‐C and ‐E gene activities in whorl 4 specify carpel identity.

Most class‐A, ‐B, ‐C and ‐E floral organ identity genes encode MADS‐domain transcription factors that act in higher order protein complexes to regulate gene expression (Smaczniak *et al.*, 2012). Genomic studies on these transcription factors have begun to reveal the gene regulatory networks that control the development of each floral organ type (Kaufmann *et al.*, 2009; Kaufmann *et al.*, 2010; Wuest *et al.*, 2012; O’Maoiléidigh *et al.*, 2013). These studies indicate that floral organ identity proteins regulate a large number of genes throughout floral organ development, with distinct genes being regulated at different stages of organ development (as reviewed by Stewart *et al.*, 2016; Yan *et al.*, 2016). Direct regulatory targets include other transcription factors as well as genes involved in plant hormone signaling pathways and developmental processes, such as pattern formation and morphogenesis. Furthermore, the floral organ identity proteins appear to repress the expression of genes that specify a leaf developmental program (O’Maoiléidigh *et al.*, 2013).

Genetic studies have uncovered multiple roles for *ANT* and *AIL6/PLT3* during the initiation and subsequent development of floral organ primordia. *ANT* and *AIL6/PLT3* promote the initiation of floral organ primordia at defined positions within the floral meristem, and also prevent premature differentiation of both primordial and floral meristem cells (Krizek, 2009; Krizek and Eaddy, 2012). *ANT* and *AIL6/PLT3* are required for the proper expression of the class‐B and ‐C floral organ identity genes *APETALA 3* (*AP3*) and *AGAMOUS* (*AG*), respectively, and consequently for the elaboration of petal and stamen fates (Krizek, 2009). *ANT* and *AIL6/PLT3* also regulate the growth of developing floral organs, thus contributing to morphogenesis and the attainment of correct organ size (Elliott *et al.*, 1996; Klucher *et al.*, 1996; Krizek, 1999; Mizukami and Fischer, 2000). Despite their well‐established importance in many facets of flower development, we currently know very little about the downstream target genes that are activated or repressed by ANT and AIL6/PLT3. Without this knowledge, we lack understanding of the biological processes regulated by these transcription factors that contribute to the sculpting of each floral organ type.

A genomic study comparing the transcriptomes of the wild type and the *ant ail6* double mutants identified thousands of differentially expressed (DE) genes, consistent with the numerous roles of ANT and AIL6/PLT3 in floral organogenesis and the severe phenotypic consequences of losing *ANT* and *AIL6/PLT3* functions (Krizek *et al.*, 2016). This experiment suggested that ANT and AIL6/PLT3 functions are intimately coupled within a vast transcriptional network regulating floral organogenesis. To distinguish between short‐term and longer‐term consequences of loss of *ANT* and *AIL6/PLT3* activities, we have adopted more focused genomic studies. We performed two complementary experiments: RNA‐Seq analysis of floral buds at 2, 4 and 8 h after the induction of ANT activity, and chromatin immunoprecipitation followed by sequencing (ChIP‐Seq) analysis of stage‐6/7 flowers. Together, these studies identified 200 genes that are both differentially expressed after ANT induction and are bound by ANT and are thus likely to be direct targets of ANT regulation. Our experiments suggest that ANT controls floral organogenesis through the direct regulation of growth and patterning genes as well as auxin responses.

## RESULTS

### RNA‐Seq identifies genes differentially expressed after the activation of ANT‐GR

To identify direct targets of ANT regulation, we created a line of transgenic plants containing an inducible form of ANT in which the ligand‐binding domain of the glucocorticoid receptor (GR) was fused to the coding region of *ANT*. In this system, the GR domain blocks migration of the ANT‐GR fusion partner into the nucleus, rendering it incapable of activating or repressing the expression of target genes. Applying the steroid dexamethasone (dex) causes a conformational change in GR that permits entry of ANT‐GR into the nucleus, where it can bind to target promoters and regulate gene expression. Applying dex to *35S:ANT‐GR* inflorescences led to the production of larger flowers and caused male sterility, similar to the phenotypes observed in *35S:ANT* inflorescences (Figure S1) (Krizek, 1999; Yamaguchi *et al.*, 2013). This established that the ANT‐GR line contained inducible ANT activity that could be triggered by the application of dex.

**Figure 1:**
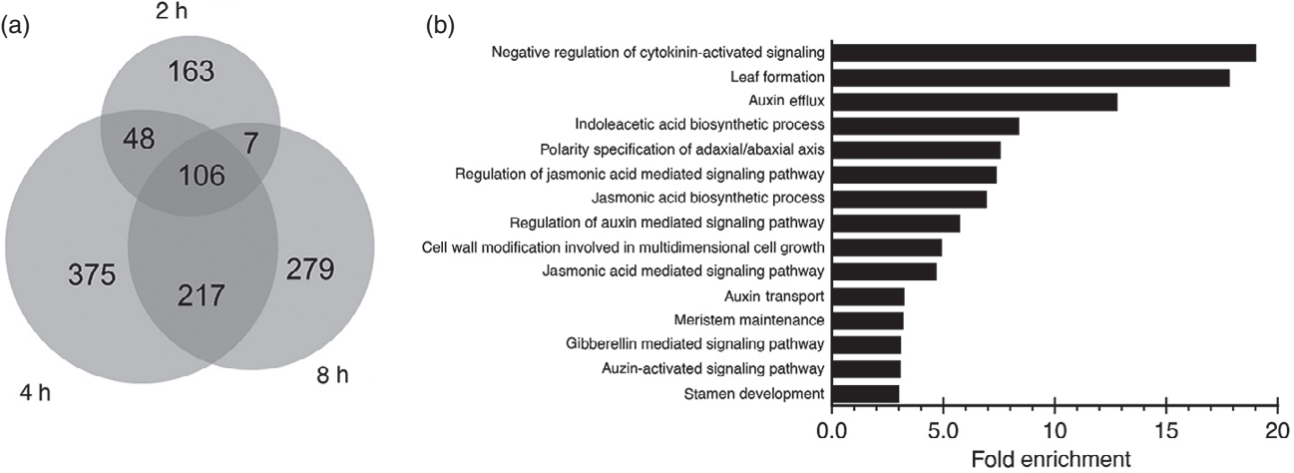
Genes differentially expressed (DE) in mock‐ and dex‐treated *35S:ANT‐GR* inflorescences. (a) Venn diagram showing the overlap between DE genes identified at 2, 4 and 8 h after treatment. The Venn diagram was created with biovenn (Hulsen *et al.*, 2008). (b) Biological process gene ontology (GO) terms enriched in DE genes.

We performed a time‐course experiment using the *35S:ANT‐GR* line, in which floral buds were collected 2, 4 and 8 h following treatment with dex or treatment with solvent only (mock) as a negative control. Whole inflorescences corresponding to floral buds at stages 1–12 were collected as four matched pairs of treatment and control samples and processed for RNA sequencing. Following sequencing and alignment to the Arabidopsis reference genome, each sample yielded between 13 and 29 million aligned fragments, which were then analyzed for differential expression. Using a false discovery rate (FDR) of 0.05, we identified 1195 genes that were DE at one or more time points (Appendix S1). More DE genes were present at 4 h (746) and 8 h (609) after treatment, compared with 2 h (324) after treatment (Figure 1a). The log2 fold change (logFC) values of the DE genes ranged from 3.15 to −1.80. There were 106 genes that were DE at all three time points. Approximately equal numbers of upregulated (836) and downregulated (843) genes were identified.

To visualize gene expression changes over time, we developed an interactive r shiny app (called show gene expression) that plots individual gene expression data over the time course of the experiment (https://bitbucket.org/krizeklab) (Figure S2a–d). The app is available in the inducible‐ant‐rna‐seq repository (/DifferentialExpression/ShowGeneExpression). After launching the shiny app in the rstudio application, users can generate plots showing the expression of a gene of interest after entering its gene identifier. Gene expression can be visualized as: ‘Sample RPKM’, with individual treatment and time points selected; ‘Group RPKM’, which averages the four replicates for each sample; or ‘Expression over time’, in which the data can be visualized with lines, points or arrows that indicate up‐ or downregulation between the control and treated samples. The ‘gene info’ tab provides a link to The Arabidopsis Information Resource (TAIR, https://www.arabidopsis.org) for further information about the gene. We used this app to investigate the expression of candidate target genes and explore their functions.

### Differentially expressed genes were enriched in terms associated with development and hormone physiology

We used enrichment analysis to identify gene ontology (GO) and Kyoto encyclopedia of genes and genomes (KEGG) functional annotation categories over‐represented among the 1195 DE genes. We further investigated individual gene functions using TAIR for members of these categories.

Many DE genes had functions related to lateral organ development, consistent with the role of ANT as a regulator of organogenesis. These included: leaf formation (GO:0010338); polarity specification of adaxial/abaxial axis (GO:0009944); and stamen development (GO:0048443) (Figure 1b). Seven genes that regulate lateral organ polarity were differentially expressed (Table S1). These genes included: three *YABBY* genes – *CRABS CLAW* (*CRC*), *YAB3* and *YAB5*; one class‐III HD‐ZIP gene – *PHABULOSA* (*PHB*); one *KANADI* gene – *KAN2*; *ASYMMMETRIC LEAVES 1* (*AS1*); and *BLADE ON PETIOLE 1* (*BOP1*). Thus, both adaxial (*PHB*, *AS1* and *BOP1*) and abaxial (*CRC*, *YAB3*, *YAB5* and *KAN2*) genes were identified as potential targets of ANT regulation. The identification of genes regulating adaxial/abaxial axis specification is consistent with previous genetic work suggesting that ANT contributes to organ polarity (Nole‐Wilson and Krizek, 2006). Leaf development GO category genes include several genes regulating organ growth, including *KLUH* (*KLU/CYP78A5*), *GROWTH REGULATING FACTORS* (*GRF3*, *GRF6* and *GRF8*) and *ANGUSTIFOLIA 3/GRF1‐INTERACTING FACTOR 1* (*AN3/GIF1*).

Several genes with known roles in floral organ development were DE. These included floral organ identity genes *APETALA 1* (*AP1*), *APETALA 2* (*AP2*) and *SEPALLATA 3* (*SEP3*), as well as genes that function in later aspects of stamen development [*SQUAMOSA PROMOTER‐BINDING PROTEIN‐LIKE 8* (*SPL8*), *EXCESS MICROSPOROCYTES 1* (*EMS1*)] and carpel development [*SPATULA* (*SPT*), *CRC*, *STRUBBELIG* (*SUB*)] (Table S1).

In addition to genes involved in lateral organ development, seven DE genes involved in meristem maintenance (GO:0010073) were identified: *CORYNE* (*CRN*), *BARELY ANY MERISTEM 3* (*BAM3*), *CLAVATA3 INSENSITIVE RECEPTOR KINASE 4* (*CIK4*), *MINI ZINC FINGER 2* (*MIF2*) and *FANTASTIC FOUR 1*, *2* and *3* (*FAF1*, *FAF2* and *FAF3*) (Table S1). This suggests that ANT has a role in meristem maintenance, in addition to its role in lateral organ development, consistent with genetic work showing the importance of *ANT* and *AIL6/PLT3* in preventing the premature differentiation of floral meristem cells (Krizek and Eaddy, 2012).

Many hormone‐related functions were enriched in the DE genes (Appendix S2), suggesting that ANT function is closely linked with multiple aspects of hormone physiology. These GO terms included: negative regulation of the cytokinin‐activated signaling pathway (GO:0080037); regulation of the jasmonic acid‐mediated signaling pathway (GO:2000022); regulation of the auxin‐mediated signaling pathway (GO:0010928); regulation of the jasmonic acid‐mediated signaling pathway (GO:0009867); regulation of the gibberellin‐mediated signaling pathway (GO:0010476); regulation of the auxin‐activated signaling pathway (GO:0009734); hormone biosynthesis – indoleacetic acid biosynthetic process (GO:000009684) and jasmonic acid biosynthetic process (GO:0009695); and hormone transport – auxin efflux (GO:0010315) and auxin transport (GO:0060918) (Figure 1b). Additional analysis using the KEGG pathway framework also found links with cytokinin, auxin, and gibberellin hormone signaling pathways (Figure S3). DE genes associated with the synthesis/metabolism, transport, signaling and response for cytokinin, auxin, gibberellin, abscisic acid and jasmonic acid (JA) are shown in Table S2. These results suggest that ANT plays roles in the metabolism of auxin and JA (and perhaps cytokinin and gibberellin) while also influencing signaling pathways downstream of cytokinin, auxin, gibberellin, abscisic acid and JA. Our earlier RNA‐Seq investigation of *ant ail6* double mutants inflorescences previously linked ANT function with auxin biosynthesis and JA signaling, but not with cytokinin or gibberellin signaling (Krizek *et al.*, 2016).

### ChIP‐Seq identifies ANT genomic binding sites in stage‐6/7 flowers

To identify genome‐wide ANT binding sites, we performed chromatin immunoprecipitation in combination with next‐generation sequencing (ChIP‐Seq). For these studies, we used a transgenic line in which an *ANT‐VENUS* gene fusion was expressed under the control of the *ANT* promoter in the *ant* mutant background. Fusion of ANT with VENUS, a rapidly folding variant of YELLOW FLUORESCENT PROTEIN (YFP), allows the immunoprecipitation of DNA bound to ANT by a commercially available antibody against GREEN FLUORESCENT PROTEIN (GFP). The transgene fully rescued the *ant‐4* mutant phenotype, as assayed by petal measurements and floral organ counts, indicating that the ANT‐VENUS fusion protein has full ANT activity (Tables S3, S4). The *ANT:ANT‐VENUS ant‐4* line was crossed into a genetic background in which flower development can be synchronized (i.e. *AP1:AP1‐GR ap1 cal*) (O’Maoiléidigh *et al.*, 2015), allowing us to collect large numbers of flowers of the same developmental stage. Inflorescences from two biological replicates of *AP1:AP1‐GR ap1 cal* and *AP1:AP1‐GR ap1 cal ANT:ANT‐VENUS ant‐4* were harvested 5 days after dex treatment, when they are composed of stage‐6/7 flowers.

**Figure 2:**
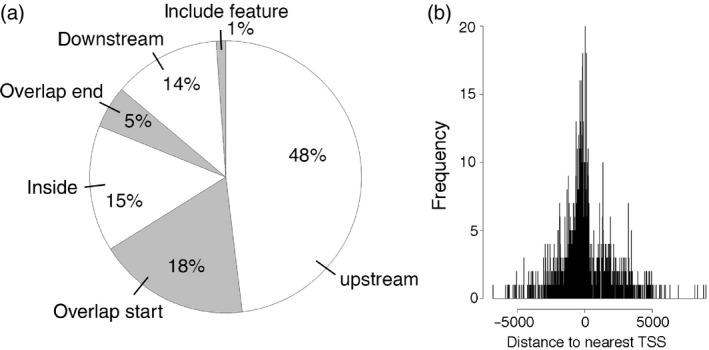
Position of ANT ChIP‐Seq peaks relative to the closest gene. (a) Pie chart showing the position of ANT ChIP‐Seq binding peaks relative to the closest gene. Almost half of the peaks are upstream of the closest gene (48%). The remaining peaks either overlap with the start of the gene (18.0%), are within the gene (15%), overlap with the end of the gene (5%), are downstream of the gene (14%) or overlap the entire gene (1%). (b) Position of ANT binding peak relative to the transcriptional start site (TSS) of the closest gene.

In our ChIP‐Seq experiment, we identified 1113 ANT binding peaks in stage‐6/7 flowers using visual analytics and the integrated genome browser (igb). These peaks were associated with 1081 unique genes (Appendix S3). Almost half of the peaks (48%) are present upstream of the gene near the transcriptional start site (TSS), with the remaining peaks overlapping the start of transcription (18%), sitting within the gene (15%), overlapping the end of transcription (5%), sitting downstream of the gene (14%) or encompassing the gene (1%) (Figure 2a,b).

### Genes associated with ANT binding sites include regulators of polarity specification, floral organ development and meristem development

**Figure 3:**
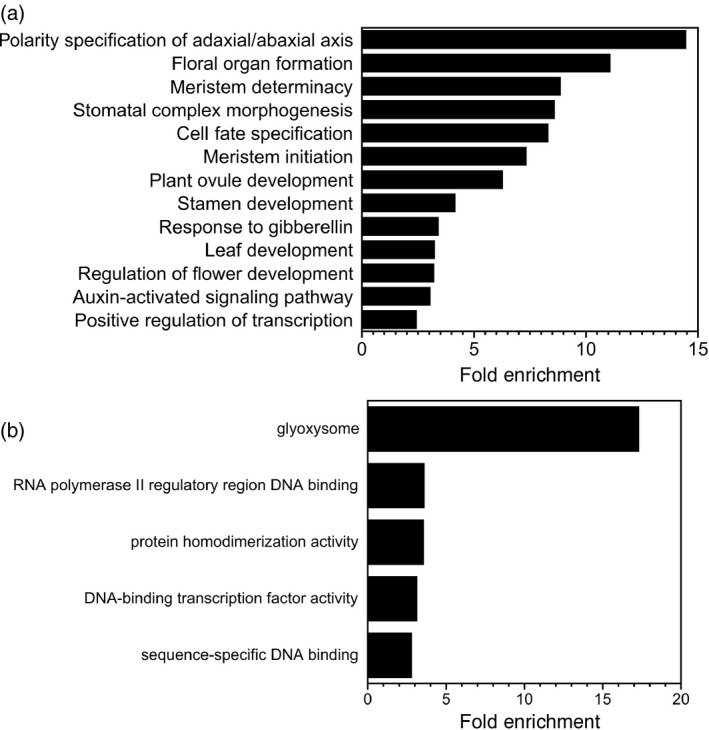
Gene ontology (GO) enrichment analyses on genes associated with ANT ChIP‐Seq binding peaks. (a) Biological process GO terms enriched in genes associated with ANT binding peaks. (b) Molecular function and cellular component GO terms enriched in genes associated with ANT binding peaks.

To gain insight into the set of genes associated with ANT ChIP‐Seq peaks, we performed a GO enrichment analysis (Appendix S4; Figure 3). Several of the identified GO terms were the same or similar to those identified in the ANT‐GR RNA‐Seq experiment. Within the biological process GO category, a number of developmental terms were identified that relate to adaxial/abaxial polarity, floral organ development and meristem development, including the following: polarity specification of adaxial/abaxial axis (GO:0009944); floral organ formation (GO:0048449); meristem determinacy (GO:0010022); meristem initiation (GO:0010014); plant ovule development (GO:0048481); stamen development (GO:0048443); and regulation of flower development (GO:0009909) (Figure 3a). Other over‐represented developmental GO biological process terms include: stomatal complex morphogenesis (GO:0010103); cell fate specification (GO:0001708); and leaf development (GO:0048366) (Figure 3a). Two hormone‐related over‐represented GO biological process terms are the response to gibberellin (GO:0009739) and the auxin‐activated signaling pathway (GO:0009734), which were also identified in the RNA‐Seq experiment (Figure 3a); however, several other hormone‐related GO terms, such as those related to cytokinin and JA signaling identified in the RNA‐Seq experiment, were not enriched in the ChIP‐Seq experiment.

The biological process GO term ‘positive regulation of transcription, DNA‐templated’ (GO:0045893) was also over‐represented, which suggests that ANT regulates the expression of other transcription factors (Figure 3a). This is also supported by the identification of the following enriched molecular function GO terms: RNA polymerase II regulatory region DNA binding (GO:0001012); DNA‐binding transcription factor activity (GO:0003700); and sequence‐specific DNA binding (GO:0043565) (Figure 3b). Another enriched molecular function GO term was protein homodimerization activity (GO:0042803) (Figure 3b).

The enriched cellular component GO term glyoxysome (GO:0009514) was also identified (Figure 3b). This category includes two enzymes, isocitrate lyase (ICL) and malate synthase (MS), specific to the glyoxylate cycle that occurs in glyoxysomes.

### Two hundred DE genes bound by ANT are likely to be direct targets of ANT regulation

**Figure 4:**
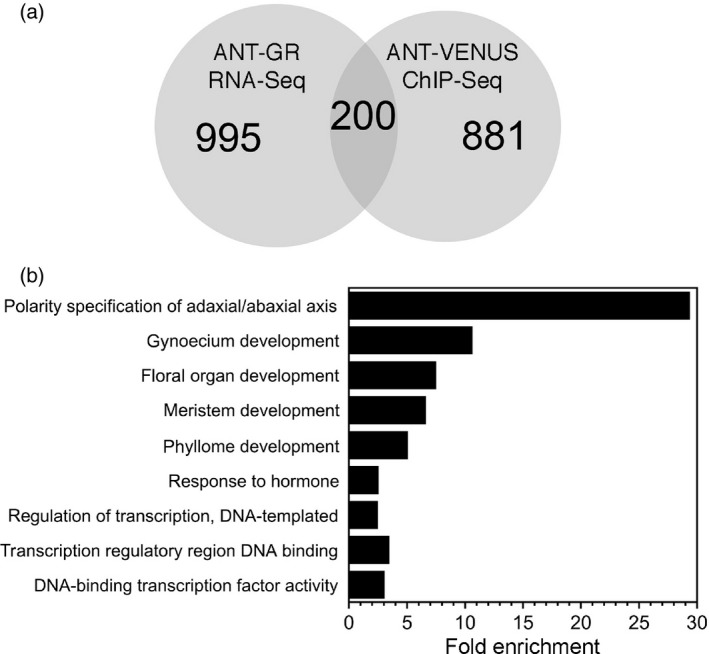
Differentially expressed (DE) genes that are bound by ANT. (a) Venn diagram showing the overlap between the DE genes identified and those bound by ANT. The Venn diagram was created with biovenn (Hulsen *et al.*, 2008). (b) Biological process and molecular function GO terms enriched in genes that are both DE and bound by ANT.

The most likely direct targets of ANT regulation are genes that are both bound by ANT and are differentially expressed in response to changes in ANT activity. There were 200 genes shared between the set of RNA‐Seq DE genes and the set of ChIP‐Seq bound genes (Figure 4a; Appendix S5). Although the total set of DE genes (1195) consists of slightly less upregulated genes (523) than downregulated genes (672), a much larger number of upregulated genes (154) are associated with ANT binding peaks as compared with downregulated genes (46).

Gene ontology (GO) term enrichment analysis on this set of 200 genes identified several GO terms similar to, or the same as, those identified for the entire set of ANT ChIP‐Seq associated genes (Appendix S6; Figure 4). In particular, three terms were enriched in both sets: polarity specification of adaxial/abaxial axis (GO:0009944); regulation of transcription, DNA‐templated (GO:0006355); and DNA‐binding transcription factor activity (GO:0003700) (Figure 4). Five polarity genes were identified: *PHB*, *BOP1*, *AS1*, *KAN2* and *YAB3* (Table 1). Other enriched GO terms were the following: gynoecium development (GO:0048467); floral organ development (GO:0048437); meristem development (GO:0048507); phyllome development (GO:0048827); and response to hormone (GO:0009725). Floral organ development genes include those involved in specifying floral organ identity (*AP1*, *AP2* and *SEP3*), regulating cellular differentiation (*EMS1* and *SPL8*) and controlling morphogenesis (*SUB* and *SPT*) (Table 1). Nine auxin genes were part of the hormone responses category, including those involved in biosynthesis (*TAA1*), signaling (*AFB2*, *ARF6*, *ARF11*, *ARF18*, *IAA3/SHY2*, *IAA27* and *PAP2*) and responses (*SAUR50* and *SAUR14*) (Table 2).

**Table 1:** Developmental genes that are differentially expressed after ANT‐GR activation and are bound by ANT

AGI locus code	Gene	log2 fold change (h)	ChIP‐Seq
Polarity specification			
AT2G34710	*PHB*	0.256 (4); 0.346 (8)	Upstream; overlap start
AT3G57130	*BOP1*	−0.449 (4)	Upstream
AT2G37630	*AS1*	−0.235 (4); −0.175 (8)	Upstream
AT1G32240	*KAN2*	0.227 (4); 0.255 (8)	Inside
AT4G00180	*YAB3*	0.269 (2); 0.262 (4); 0.374 (8)	Upstream
Floral organ development			
AT1G69120	*AP1*	−0.144 (4)	Upstream; overlap start
AT4G36920	*AP2*	0.22 (2)	Upstream
AT1G24260	*SEP3*	0.175 (8)	Inside
AT1G02065	*SPL8*	−0.341 (2); −0.589 (4); −0.534 (8)	Overlap start
AT5G07280	*EMS1*	−0.14 (4)	Upstream
AT1G11130	*SUB*	0.154 (4); 0.165 (8)	Overlap start
AT4G36930	*SPT*	0.269 (2); 0.297 (4); 0.418 (8)	Upstream
Growth genes			
AT1G13710	*KLU/CYP78A5*	−0.281 (2); −0.272 (4)	Upstream
AT4G24150	*GRF8*	0.317 (8)	Inside
AT5G28640	*AN3/GIF1*	0.244 (2); 0.25 (4); 0.403 (8)	Overlap start, inside
AT4G03210	*XTH9*	0.315 (2); 0.351 (4); 0.405 (8)	Upstream
AT2G47780	*SRP2*	0.482 (4); 0.718 (8)	Overlap start
AT2G32710	*KRP4*	0.293 (2); 0.299 (4); 0.331 (8)	Overlap end

**Table 2:** Hormone genes that are differentially expressed after ANT‐GR activation and are bound by ANT

AGI locus code	Gene	log2 fold change (h)	ChIP‐Seq
Auxin			
AT1G70560	*TAA1/WEI8*	0.297 (4); 0.444 (8)	Inside
AT3G26810	*AFB2*	0.185 (8)	Upstream
AT1G30330	*ARF6*	0.132 (4)	Upstream
AT2G46530	*ARF11*	0.376 (8)	Upstream
AT3G61830	*ARF18*	0.198 (8)	Overlap end
AT1G04240	*SHY2/IAA3*	0.285 (8)	Overlap start
AT4G29080	*PAP2/IAA27*	0.14 (4)	Upstream
AT4G34760	*SAUR50*	0.346 (8)	Upstream
AT4G38840	*SAUR14*	−0.478 (4)	Overlap start
Gibberellin			
AT1G14920	*GAI*	0.133 (4); 0.181 (8)	Upstream
AT2G01570	*RGA*	0.246 (2); 0.259 (4); 0.334 (8)	Upstream
Brassinosteroid			
AT1G78700	*BEH4*	0.664 (2); 0.734 (4); 0.918 (8)	Inside
Abscisic acid			
AT2G40330	*PYL6*	0.552 (2)	Overlap start
Ethylene			
AT1G15360	*WIN1/SHN1*	−0.212 (8)	Overlap start

To confirm our RNA‐Seq and ChIP‐Seq results, we selected eight genes from this set of 200. The selected genes were not previously known to be regulated by ANT and were associated with several different biological processes. The eight genes included: two polarity genes, *KAN2* and *PHB*; two hormone signaling genes, *BES1/BZR1 HOMOLOG 4* (*BEH4*) and *REPRESSOR OF GA* (*RGA*); the reproductive organ development gene *SPL8*; and three genes that regulate lateral organ growth, *AN3/GIF1*, *XYLOGLUCAN ENDOTRANSGLUCOSYLASE/HYDROLASE 9* (*XTH9*) and *SMALL RUBBER PARTICLE PROTEIN2* (*SRP2*) (Table 1).

**Figure 5:**
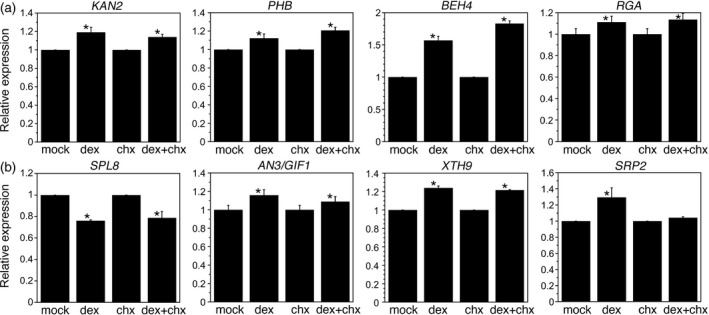
Several differentially expressed (DE) genes tested show protein synthesis‐independent gene expression changes after induction of ANT activity in *35S:ANT‐GR*. Graphs show the relative expression of *KAN2, PHB*, *BEH4, RGA, SPL8*, *AN3/GIF1*, *XTH9* and *SRP2* after mock, dexamethasone (dex), cycloheximide (chx) and dex + chx treatments. Relative expression refers to mRNA levels in dex samples compared with mock samples and mRNA levels in dex + chx samples compared with chx samples. *The dex samples statistically differ from mock samples and the dex + chx samples statistically differ from chx samples, as determined by Students *t*‐test (*P* < 0.05). mRNA levels were measured 4 h after treatment. Graphs show means ± SDs of two biological replicates.

We examined the expression of these eight genes in an independent batch of mock‐ and dex‐treated *35S:ANT‐GR* inflorescences. To determine whether the activation of these target genes required novel protein synthesis, we included inflorescences treated with the protein synthesis inhibitor cycloheximide (chx). Inflorescences were collected 4 h after the respective treatment. Similar changes in gene expression in inflorescences treated with dex + chx versus chx alone, as compared with those treated with dex versus mock, support the direct regulation of the gene by ANT. Seven of the eight DE genes showed expression changes independent of protein synthesis in *35S:ANT‐GR* inflorescences (Figure 5). The lone exception was *SRP2*, which showed similar expression levels in chx and dex + chx samples (Figure 5). Thus, SRP2 may not be a direct target of ANT regulation, although it is possible that the use of whole inflorescences in the quantitative reverse transcription PCR (RT‐qPCR) experiment obscured developmental stage‐specific regulation of this gene by ANT in stage‐6/7 flowers.

**Figure 6:**
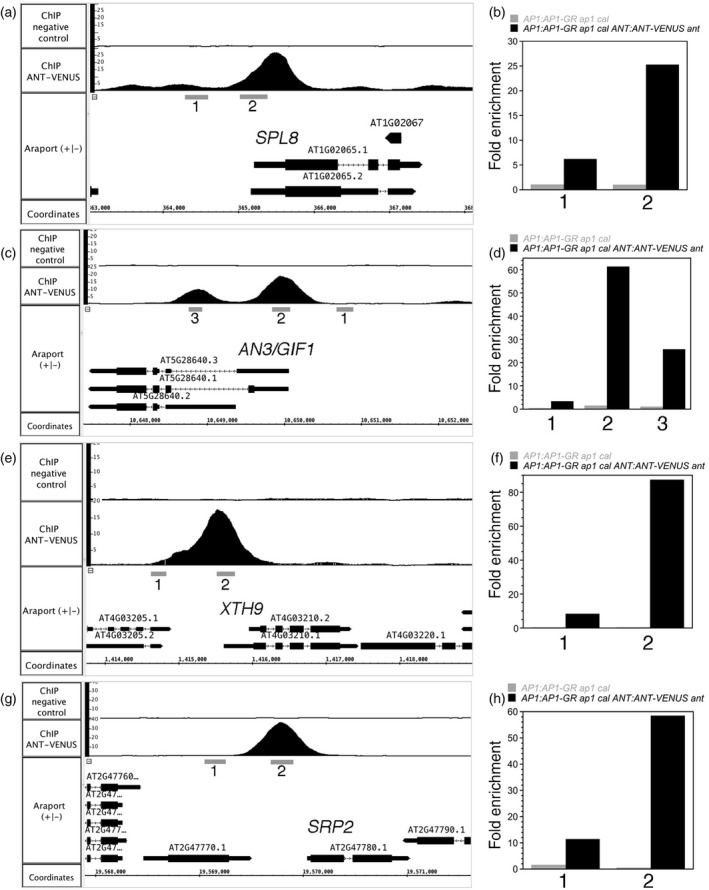
ChIP‐qPCR confirms that ANT binds to genomic regions upstream or within genes associated with stamen development (*SPL8*) and growth (*AN3/GIF1*, *XTH9*, *SRP2*). ChIP‐Seq coverage graphs for *SPL8* (a), *AN3/GIF1* (b), *XTH9* (e), and *SRP2* (g). Numbers below the gene indicate the regions tested for ANT binding by ChIP‐qPCR. ChIP‐qPCR data for *SPL8* (b), *AN3/GIF1* (d), *XTH9* (f) and *SRP2* (h). Grey bars show results from *AP1:AP1‐GR ap1 cal* and black bars show results from *AP1:AP1‐GR ap1 cal ANT:ANT‐VENUS ant*. Numbers on the x axis correspond to the genomic regions indicated in the ChIP‐Seq coverage graphs.

The ChIP‐qPCR experiments performed on these eight genes with an independent batch of ChIP DNA gave results that are similar to those seen by ChIP‐Seq (Figures 6 and S4). ANT binds to regions upstream or overlapping the 5' untranslated region (5'‐UTR) (*PHB*, *RGA*, *SPL8*, *AN3/GIF1*, *XTH9* and *SRP2*) or within the gene body (*KAN2* and *BEH4*) in stage‐6/7 flowers. No enrichment was observed in *AP1:AP1‐GR ap1 cal* inflorescences lacking *ANT:ANT‐VENUS*. ANT binds to genomic regions of both upregulated (*KAN2*, *PHB*, *BEH4*, *RGA*, *AN3/GIF1*, *XTH9* and *SRP2*) and a downregulated (*SPL8*) gene.

### DNA sequence motif analyses of ChIP‐Seq peaks

In previous work using SELEX, we identified the *in vitro* DNA binding motif of ANT (Nole‐Wilson and Krizek, 2000). To determine whether ANT binding peaks contain DNA sequences with similarity to this motif, we mapped putative ANT binding sites on a genome‐wide level using the fimo program from the meme software suite (Grant *et al.*, 2011). We then compared the position of the fimo‐predicted sites to both the ChIP‐Seq peaks and to a set of randomized peaks of the same size. About 66.7% of the ChIP‐Seq peaks and 43.8% of the randomized peaks overlapped with a fimo site. Thus, ChIP‐Seq peaks were more likely than randomized peaks to contain a fimo‐predicted ANT binding site; however, nearly a third of the ChIP‐Seq peaks did not contain a fimo‐predicted ANT binding site.

**Figure 7:**
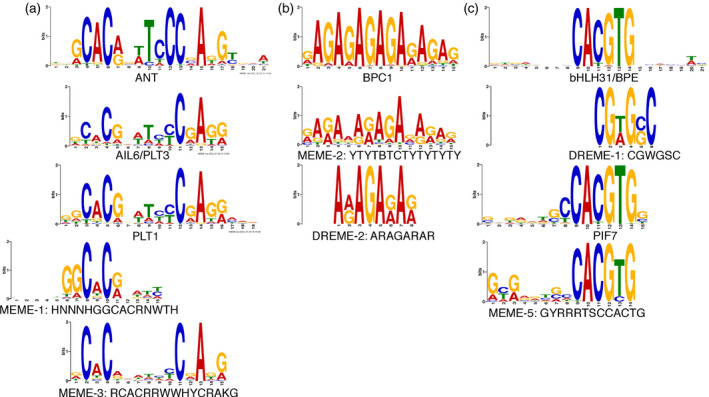
meme‐chip identifies sequences with similarity to AIL/PLT sites as well as other transcription factors. (a) Sequence logos representing the DNA binding specificities of ANT, AIL6/PLT3, PLT1 and two motifs (MEME‐1 and MEME‐3) identified within ANT ChIP‐Seq binding peaks. (b) Sequence logos representing the DNA binding specificity of BPC1 and two related motifs (MEME‐2 and DREME‐2) identified within ANT ChIP‐Seq binding peaks. (c) Two bHLH binding sites from bHLH31/BPE and PIF7 and two related motifs (DREME‐1 and MEME‐5) identified within ANT ChIP‐Seq binding peaks.

We used meme‐chip from the meme suite to perform novel motif discovery (Machanick and Bailey, 2011). Our analysis used the DAP‐Seq database for motif discovery, which includes several AIL/PLT binding sites but not that of ANT (O’Malley *et al.*, 2016). MEME‐ChIP identified seven motifs with an e‐value of 1.00E–10 or lower (Table 3). Two of these motifs, MEME‐1 (HNNNHGGCACRNWTH) and MEME‐3 (RCACRRWWHYCRAKG), were similar to the PLT1 and AIL6/PLT3 DAP‐Seq binding motifs, respectively (Figure 7a; Table 3). The PLT1 and AIL6/PLT3 binding sites consisted of a fairly long sequence with several conserved residues near each end of the site and fewer conserved nucleotides in the center. The ANT SELEX‐determined *in vitro* binding motif is similar to both of these sites (Figure 7a) (Nole‐Wilson and Krizek, 2000). The MEME‐1 motif had similarity to the first conserved part of the AIL/PLT sites, whereas the MEME‐3 motif had similarity to both conserved parts of AIL/PLT sites (Figure 7a). Thus, the identification of MEME‐1 and MEME‐3 motifs in ANT binding peaks suggests that the *in vivo* binding specificity of ANT resembles that determined *in vitro*, but with reduced conservation at several positions within the motif. Interestingly, in some MEME‐1 sites, a second motif was identified at a conserved distance from the MEME‐1 motif and resembled the second half of the AIL/PLT binding sites (Figure S5).

**Table 3:** meme‐chip analysis of ANT ChIP‐Seq peaks

Motif	Motif ID	Width	Sites	*e*‐value	Most similar motif
HNNNHGGCACRNWTH	MEME‐1	15	267	5.80E‐251	PLT1 (AP2/ERF)
YTYTBTCTYTYTYTY	MEME‐2	15	400	2.00E‐169	BPC5 (BBR/BPC)
RCACRRWWHYCRAKG	MEME‐3	15	263	7.30E‐99	AIL6/PLT3 (AP2/ERF)
CGWGSC	DREME‐1	6	221	8.70E‐19	BPE/bHLH31 (bHLH)
ARAGARAR	DREME‐2	8	348	5.30E‐16	BPC1 (BBR/BPC)
AAARGHRGARARARAAADARAAVAAMAAA	MEME‐4	29	64	2.60E‐15	VRN1 (ABI3/VP1)
GYRRRTSCCACGTG	MEME‐5	14	39	8.30E‐11	PIF7 (bHLH)

meme‐chip identified several other enriched motifs in ANT binding peaks. These had similarity to the binding sites of other transcription factors. Two such motifs, MEME‐2 (YTYTBTCTYTYTYTY) and DREME‐2 (ARAGARAR), resembled the binding sites of BASIC PENTACYSTEINE (BPC) transcription factors (Figure 7b; Table 3). These transcription factors bind GA repeat sequences present in many plant promoters. Recently, BPC proteins were shown to bind to Polycomb response elements (PREs) and interact with components of Polycomb repressive complex 2 (PRC2) to mediate the silencing of gene expression by PRC2 (Xiao *et al.*, 2017). Two additional motifs, MEME‐5 (GYRRRTSCCACGTG) and DREME‐1 (CGWGSC), resembled the binding sites of basic helix–loop–helix (bHLH) transcription factors (Figure 7c; Table 3). In particular, the identified motifs most closely match the DAP‐Seq binding sites of BIG PETAL (BPE)/bHLH31 and PHYTOCHROME INTERACTING FACTOR 7 (PIF7).

### The ANT ChIP‐Seq gene set exhibited limited overlap with those from AP1, JAG and PLT2

To investigate where ANT acts within the hierarchy of known flower development regulators, we compared the set of genes bound by ANT to genes bound by other transcription factors involved in flower development, as determined by ChIP‐chip or ChIP‐Seq experiments. We compared ANT with the floral meristem identity protein LFY, the floral organ identity proteins AP1, AP3, PI, AG and SEP3, the growth regulator JAGGED (JAG) and AUXIN RESPONSE FACTOR 3/ETTIN (ARF3/ETT), which regulates gynoecium development (Winter *et al.*, 2011; Wuest *et al.*, 2012; O’Maoiléidigh *et al.*, 2013; Pajoro *et al.*, 2014; Schiessl *et al.*, 2014; Simonini *et al.*, 2017). Fisher’s exact tests were performed using the r package geneoverlap (Shen and Sinai, 2019). geneoverlap also calculates the Jaccard index to assess the overlap of two gene lists. A Jaccard index of 0 indicates no similarity between the gene lists whereas a value of 1 indicates that the lists are identical. Comparisons between the gene set of ANT with gene sets from LFY, AP3, PI, AG, SEP3 and ETT each gave a Jaccard index of 0.1, whereas the comparison between ANT and JAG and that between ANT and AP1 gave a Jaccard index of 0.2 (Figure S6). This degree of overlap is much less than that observed among the floral organ identity proteins AP3, PI and AG, which exhibit Jaccard indices of 0.4 or 0.5 (Figure S6). JAG also showed a Jaccard index of 0.2 with the floral organ identity proteins SEP3, AP1 and PI.

We also compared the set of ANT ChIP‐Seq bound genes with genes bound by two other AIL/PLT transcription factors: PLT2, which specifies stem cell identity in the root and regulates shoot phyllotaxy; and BABYBOOM (BBM), which promotes somatic embryogenesis (Boutilier *et al.*, 2002; Aida *et al.*, 2004; Prasad *et al.*, 2011). The PLT2 ChIP‐Seq experiment used roots and the BBM ChIP‐Seq experiment used somatic embryos (BBM) (Horstman *et al.*, 2015; Santuari *et al.*, 2016). The comparison with PLT2 gave a Jaccard index of 0.2, whereas the comparison with BBM gave a Jaccard index of 0.1. It is interesting that ANT showed more overlap with the related PLT2 transcription factor, which primarily functions in roots, compared with most non‐related transcription factors that regulate floral organ identity.

### ANT may repress *SPL8* to promote petal growth

**Figure 8:**
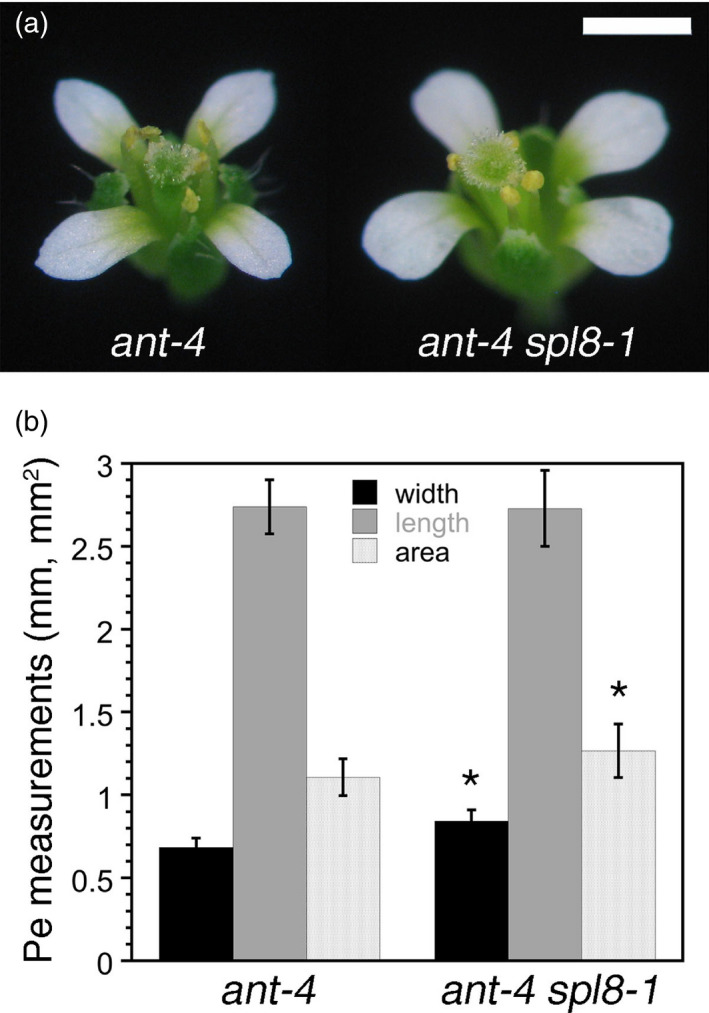
Mutations in *SPL8* partially rescue the petal size defects of *ant* flowers. (a) *ant‐4* flower (left) and *ant‐4 spl8‐1* flower (right). (b) Petal width, length and area in *ant‐4* and *ant‐4 spl8‐1* flowers. *P < 0.05 (Student's *t*‐test). Scale bar: 1 mm.

One of the 200 likely direct targets of ANT regulation is *SPL8*, a gene that acts in micro‐ and megasporogenesis (Unte *et al.*, 2003). *spl8* mutants produce slightly thinner flowers than the wild type and have stamens with smaller anthers and shorter filaments (Unte *et al.*, 2003). *SPL8* mRNA levels are reduced in dex‐treated *35S:ANT‐GR* inflorescences, and ANT binds to its 5'‐UTR (Figures 5 and 6). This suggests that ANT can directly repress *SPL8* expression. To investigate a possible genetic interaction between *ANT* and *SPL8*, we generated *ant‐4 spl8‐1* double mutants. *ant‐4 spl8‐1* flowers exhibit a partial suppression of the petal growth defects of *ant‐4* (Figure 8a). Petal width and petal area are larger in *ant‐4 spl8‐1* flowers, compared with *ant‐4* flowers (Figure 8b). This finding suggests that *SPL8* acts as a repressor of petal growth in the *ant‐4* background and that one means by which ANT promotes petal growth is through the downregulation of *SPL8*. A role in petal development has not previously been noted for *SPL8*, although the gene is expressed in the margins of petals in stage‐8 flowers (Unte *et al.*, 2003). The molecular mechanism by which *SPL8* may repress petal growth in *ant* mutants is not clear, as *spl8‐1* mutants have smaller petals than those in wild‐type flowers (Figure S7).

## DISCUSSION

*ANT* is a key regulator of floral organ growth that acts in a redundant manner with *AIL6/PLT3* to regulate the initiation, identity and patterning of floral organs (Elliott *et al.*, 1996; Klucher *et al.*, 1996; Krizek, 1999; Mizukami and Fischer, 2000; Krizek, 2009). Using transcriptional profiling and genome‐wide mapping of ANT binding sites, we identified 200 genes that both responded to changes in ANT activity and were bound by ANT. This set of genes included auxin signaling genes as well as genes that specify floral organ identity, establish polarity, regulate growth and promote cell differentiation. Thus, our work begins to reveal the molecular means by which ANT regulates growth and patterning during floral organogenesis and identifies candidate direct target genes in the larger transcriptional network surrounding ANT.

Our studies reveal that ANT can act directly as both a transcriptional activator and a transcriptional repressor. The set of 200 likely direct targets of ANT regulation include both upregulated and downregulated genes. More upregulated genes (154) are associated with ANT binding peaks compared with downregulated genes (46), suggesting that ANT primarily acts as a transcriptional activator. Although our work suggests that ANT binds to DNA sequences *in vivo* with a similar sequence specificity as that *in vitro* not all ANT binding peaks contain an AIL/PLT‐like site, as defined by the MEME‐1 and MEME‐3 motifs determined with meme. Thus, ANT may bind directly to other DNA sequences and/or be recruited to different DNA sites via interaction with other transcription factors. The identification of DNA binding motifs of BPC and bHLH transcription factors within ANT peaks suggests that ANT may be recruited to some DNA sites via interaction with other classes of transcription factors.

A comparison of the set of genes bound by ANT with those bound by other floral regulators did not reveal strong overlap with any other gene set, as measured by the Jaccard index. This is consistent with ANT having functions that are distinct from transcription factors specifying floral meristem or floral organ identity. One limitation of these comparisons is the different tissues used in these studies. The LFY, JAG and ETT experiments were performed with whole inflorescences, whereas the SEP3, AP1, AP3, PI and AG experiments used stage‐5 flowers. Among the floral regulators tested, the highest overlap of ANT was seen with AP1 and JAG. JAG is a regulator of floral organ shape, which suggests that ANT and JAG may regulate some common target genes mediating floral organ growth. We also observed higher overlap with the root stem cell regulator PLT2 despite the different tissues used in the ChIP‐Seq experiments (stage‐6/7 flowers versus seedlings). The similar DNA binding specificities of ANT and PLT2 may result in the regulation of some common targets despite the distinct morphologies and functions of roots and flowers.

### ANT directly regulates genes involved in auxin signaling

Genomic studies in the root have shown that AIL/PLT transcription factors directly regulate genes involved in auxin biosynthesis and transport. Our studies here imply that ANT directly regulates auxin signaling in developing flowers (Santuari *et al.*, 2016; Zúñiga‐Mayo *et al.*, 2019). Although our RNA‐Seq experiment suggested that ANT could regulate other hormone signaling pathways, including cytokinin, gibberellin and JA, the DE genes in these categories were not bound by ANT and are thus likely to be indirect targets of ANT regulation. It is also possible that these genes may be bound by ANT at a different stage of flower development, however, as the RNA‐Seq experiment used floral buds of stage 1–12, whereas the ChIP‐Seq experiment used stage‐6/7 buds.

Auxin is linked to many aspects of flower development, including floral organ initiation, primordium growth, stamen filament elongation, pollen maturation and gynoecium patterning, several of which overlap with ANT function (Cheng *et al.*, 2006; Marsch‐Martînez and de Folter, 2016). Patterning of distinct tissues and cell types within the gynoecium appears to involve complex transcriptional networks and the precise distribution of hormones, particularly auxin and cytokinin (Zúñiga‐Mayo *et al.*, 2019). Within the gynoecium, *ANT* acts redundantly with several other genes including *REV* to promote the development of the carpel marginal meristem, a meristematic region within the medial domain of the ovary that gives rise to multiple tissues, including the placentae, ovules and transmitting tract (Liu *et al.*, 2000; Nole‐Wilson and Krizek, 2006; Azhakanandam *et al.*, 2008; Krizek, 2009). In *ant rev* double mutants, medial domain development is disrupted and there is partial loss of the carpel marginal meristem (Nole‐Wilson *et al.*, 2010). These defects are associated with reduced expression of the auxin biosynthetic enzyme *TAA1* in stage‐7 gynoecium (Nole‐Wilson *et al.*, 2010). We found that ANT induction activated *TAA1* expression and that ANT bound to *TAA1*. These results suggest that ANT may be a direct regulator of *TAA1* in this tissue. Mutation of the ANT binding site within the *TAA1* gene would help to reveal whether ANT is required for the expression of this gene within the gynoecium.

### ANT directly regulates genes that specify floral organ identity

Three of the 200 likely direct targets of ANT regulation were floral organ identity genes: the class‐A genes *AP1* and *AP2* and the class‐E gene *SEP3*. *AP1* was downregulated after ANT induction, whereas *AP2* and *SEP3* were upregulated (Table 1). Expression of *AP1*, *AP2* and *SEP3* is initiated early in flower development (stage 1 for *AP1* and *AP2* and stage 2 for *SEP3*) and maintained in developing flowers (Mandel *et al.*, 1992; Jofuku *et al.*, 1994; Mandel and Yanofsky, 1998). Thus, the binding of ANT to the regulatory regions of these genes in stage‐6/7 flowers may reflect a role in maintaining (*AP2* and *SEP3*) or limiting (*AP1*) expression in later stages of flower development.

The combined activities of class‐A and ‐E genes specify sepal identity, which is not disrupted in *ant* single mutants or *ant ail6* double mutants; however, in combination with class‐B genes, class‐A and ‐E genes also contribute to the specification of petal identity in the second whorl. Petals are not present in *ant ail6* flowers and expression of the class‐B gene *AP3* is reduced (Krizek, 2009; Krizek *et al.*, 2016). Although we detected binding of ANT to the regulatory regions of *AP3* in stage‐6/7 flowers (Appendix S3), we failed to observe statistically significant differential expression of *AP3* following induction of ANT activity. Failure to detect a significant difference between treatment and control samples in any time point was not a result of low *AP3* expression, as *AP3* was highly expressed in these samples. Instead, this negative result may have resulted from the timing of the floral stage examined, the bulk nature of the tissue collected or because ANT requires some other unknown factor to alter *AP3* expression.

### ANT directly regulates genes acting later in floral organogenesis that control growth and differentiation

We found that ANT directly regulates genes that act in the elaboration of organ size and shape and the differentiation of distinct cell types. We showed that ANT binds to two genes involved in stamen development: *EXCESS MICROSPOROCYTES1* (*EMS1*) and *SPL8*. EMS1 encodes a leucine‐rich repeat receptor kinase that is required for tapetal cell differentiation (Zhao *et al.*, 2002; Huang *et al.*, 2016) and *SPL8* encodes an SPB‐box transcription factor that is required for the normal development of anther sporogenic tissue (Unte *et al.*, 2003; Xing *et al.*, 2010). Both *EMS1* and *SPL8* were downregulated after induction of ANT activity, consistent with a role for ANT in inhibiting differentiation in early stages of floral organogenesis (Krizek and Eaddy, 2012).

Several known regulators of lateral organ growth were identified in the set of 200 DE genes bound by ANT. These were *KLU*, *GRF8*, *AN3/GIF1*, *XTH9* and *SRP2*, which act as growth‐promoting genes, and *KIP‐RELATED PROTEIN 4* (*KRP4*), which acts as a growth repressor (Table 1) (Hyodo *et al.*, 2003; Kim *et al.*, 2003; Kim and Kende, 2004; Anastasiou *et al.*, 2007; Bemis and Torii, 2007; Kim *et al.*, 2016). *GRF8*, *AN3/GIF1*, *XTH9* and *SRP2* were upregulated after ANT induction, suggesting that they mediate the role of ANT in promoting organ growth. Furthermore, *AN3/GIF1* and *XTH9* have largely overlapping expression patterns with *ANT*, which is consistent with ANT acting as an activator of these genes (Figure S8) (Hyodo *et al.*, 2003).

The identification of members of the GRF/GIF pathway (GRF8 and AN3/GIF) as potential targets of ANT regulation is interesting, as GRFs and their miRNA regulator *miR396* are key regulators of growth in many plant tissues (reviewed by Liebsch and Palatnik, 2020). In the root, GRF/GIF complexes repress *AIL/PLT* expression in transit‐amplifying cells to promote the proliferation of these cells, whereas PLT1 and PLT2 activate *miR396* within the root stem cell niche to repress *GRF* expression and maintain stem cell identity (Rodriguez *et al.*, 2015). The miR396‐GRF/GIF module also controls floral organ growth and meristematic competence within reproductive organs, but a connection with AIL/PLT function has not been described (Lee *et al.*, 2014; Lee *et al.*, 2018). *ant an3* double mutants exhibit more severe defects in leaf growth than either single mutant, but there was no enhancement of the carpel marginal meristem defects within the gynoecium (Lee *et al.*, 2014). Extensive genetic redundancies in the *AIL/PLT*, *GRF* and *GIF* gene families may complicate the interpretation of these results (Lee *et al.*, 2014). Future studies will need to address whether AIL/PLT might directly regulate *GRF/GIF* expression or act indirectly through *miR396* in the carpel marginal meristem, and whether *AIL/PLT* genes are targets of GRF/GIF regulation.

### ANT may promote organ growth through the direct regulation of polarity genes

Seven genes associated with lateral organ polarity were identified in our RNA‐Seq experiment, of which five genes (*PHB*, *BOP1*, *AS1*, *KAN2* and *YAB3)* were also next to an ANT binding site (Table 1). The DE genes included both upregulated and downregulated genes. We found ANT binding sites near or within four other genes that were not detected as DE. These included the adaxial genes *REVOLUTA* (*REV*) and *BOP2* and the abaxial genes *KAN2* and *FILAMENTOUS FLOWER* (*FIL*). The ANT binding peaks upstream of *FIL* and *YAB3* overlapped an ANT binding site defined *in vitro* (Nole‐Wilson and Krizek, 2006).

Previous genetic work has suggested a role for ANT in lateral organ polarity. Although *ant* single mutants do no show defects in organ polarity, mutations in *ANT* combined with mutations in *FIL* produce plants with smaller leaves that exhibit polarity defects on both the adaxial and the abaxial surfaces of leaves (Nole‐Wilson and Krizek, 2006). *ant fil yab3* triple mutants exhibit even more severe defects in leaf polarity and growth, and *YAB3* and *FIL* expression is reduced in *ant ail6* double mutants (Nole‐Wilson and Krizek, 2006; Krizek *et al.*, 2016). Polarity defects were also observed in *ant fil* floral organs, and these defects were associated with reduced expression of *PHB* (Nole‐Wilson and Krizek, 2006). Together, these data suggest that ANT regulates organ polarity through the regulation of both adaxial‐ and abaxial‐specifying genes. As the juxtaposition of adaxial and abaxial cell types is required for outgrowth of the leaf lamina, our work suggests that one mechanism by which ANT controls lateral organ growth is through the direct regulation of polarity genes to establish distinct adaxial and abaxial domains within developing lateral organ primordia (Yamaguchi *et al.*, 2012).

Overall, the work described here reveals that ANT can directly regulate the expression of target genes involved in various aspects of flower development, including floral organ identity, polarity, growth and cellular differentiation. Furthermore, our findings connect ANT function with several hormone pathways that may provide positional information for growth and patterning events during flower development.

## EXPERIMENTAL PROCEDURES

### Plant materials, growth conditions, genotyping and treatments

*35S:ANT‐GR* plants were grown on a soil mixture of Fafard 4P:perlite:vermiculite (8:1:1) in 16‐h days at a light intensity of approximately 160 µmol m^−2^ s^−1^ at 20°C. *ANT:ANT‐VENUS ant‐4 AP1:AP1‐GR ap1 cal* inflorescences were grown on a soil mixture of Fafard 4P:perlite:vermiculite (8:1:1) in 24‐h days at a light intensity of approximately 160 µmol m^−2^ s^−1^ at 20°C. *ant‐4* and *spl8‐1* were grown on a soil mixture of Fafard 4P:perlite:vermiculite (8:1:1) in 16 h days at a light intensity of approximately 160 µmol m^−2^ s^−1^ at 22°C. *ant‐4 spl8‐1* double mutants were identified by genotyping for *ant‐4* and *spl8‐1*, as described previously (Unte *et al.*, 2003; Krizek, 2009). *35S:ANT‐GR* plants for RNA‐Seq and RT‐qPCR were treated by pipetting a mock (0.1% ethanol + 0.015% Silwet), dex (10 µm dexamethasone + 0.015% Silwet), chx (10 µm cycloheximide + 0.015% Silwet + 0.1% ethanol) or dex + chx (10 µm dexamethasone + 10 µm cycloheximide + 0.015% Silwet) solution onto the inflorescences. *AP1:AP1‐GR ap1 cal* and *AP1:AP1‐GR ap1 cal ANT:ANT‐VENUS ant‐4* plants for ChIP‐Seq and ChIP‐qPCR were treated by pipetting a dex (10 µm dexamethasone + 0.015% Silwet) solution onto the inflorescences.

### RNA‐Seq

*35S:ANT‐GR* inflorescences containing unopened floral buds (flowers stages 1–12) were collected in four batches at each time point (2, 4 and 8 h after treatment) consisting of two flats per batch, where dex was applied to one flat and a mock treatment was applied to the other flat. RNA was extracted from inflorescences using Trizol following the manufacturer’s instructions with cleanup and DNase treatment on a RNeasy column (Qiagen, https://www.qiagen.com). Sequencing libraries were prepared from four biological replicates using TruSeq Stranded mRNA sample preparation kit (Illumina, https://www.illumina.com) and sequenced on the Illumina HiSeq 2500 producing 100 base single‐end reads. Sequence reads were aligned to the reference *A. thaliana* genome (version TAIR9, released June 2009) using tophat and bowtie 2. Reads per gene were counted using featurecounts. Read counts were analyzed using edger. Differentially expressed genes were identified using an additive linear model with adjustment for batch (flat) effects. Source code for differential expression analysis is available in the project ‘git’ repository https://bitbucket.org/krizeklab. GO analyses were performed with amigo 2 (http://amigo.geneontology.org/amigo).

### RT‐qPCR

RNA was isolated as described above for RNA‐Seq. First‐strand complementary DNA (cDNA) synthesis was performed using Quanta qScript cDNA SuperMix (Quanta BioSciences, https://www.quantabio.com), following the manufacturer’s instructions. qPCR was performed on a BioRad CFX96 using PerfeCTa sybr Green FastMix for iQ (Quanta BioSciences) and primers listed in Table S5. Data analyses were carried out as described previously (Krizek and Eaddy, 2012). Two biological replicates were analyzed for each experiment.

### ChIP‐Seq and ChIP‐qPCR

Chromatin immunoprecipitation was performed similarly to that described by Yamaguchi *et al.* (2014), with the same buffers and solutions. Approximately 600 mg of inflorescence tissue consisting of stage‐6/7 flowers from *AP1:AP1‐GR ap1 cal* and *ANT:ANT‐VENUS ant‐4 AP1:AP1‐GR ap1 cal* plants was collected 5 days after dex treatment into a 2‐ml tube filled with 1.5 ml of cold 1× PBS on ice. The PBS was then removed and replaced with 10 ml of 22°C 1% methanol‐free formaldehyde (ThermoFisher Scientific, https://www.thermofisher.com) in 1× PBS and 0.015% Silwet L‐77 for 15 mins at room temperature. During this time, the tissue was vacuum infiltrated three times for 2 min each time. The fixative was removed and the cross‐linking was stopped with the addition of 10 ml of 0.125 m glycine, and then incubated for 5 min. During this time, the tissue was vacuum infiltrated once for 2 min. The tissue was rinsed three times with 10 ml of cold 1× PBS on ice, dried briefly on paper towels, frozen in liquid nitrogen and then stored at −80°C. The tissue was ground in liquid nitrogen and 2.5 ml of nuclei extraction buffer with protease inhibitors and β‐mercaptoethanol was added. The samples were filtered twice through Miracloth and centrifuged at 10 000 ***g*** for 5 min at 4°C. The pellet was resuspended in 107 µl of nuclei lysis buffer and left on ice for 30 min with occasional stirring with a pipet tip. An 893‐µl volume of ChIP dilution buffer without Triton X‐100 was added to bring the volume to 1 ml. The sample was loaded into a milliTUBE 1‐ml AFA Fiber tube (Covaris, https://covaris.com) and chromatin shearing was performed with a Covaris M220 Focused ultra‐sonicator (14 cycles of 75% peak power, 5 duty factor, 200 cycles/burst at 7°C). After sonication, 200 µl of ChIP dilution buffer with Triton X‐100 and 53 µl of 22% Triton X‐100 was added to each sample. The samples were centrifuged twice at 12 000 ***g*** for 10 min at 4°C. The sample was pre‐cleared by adding 50 µl of Dynabeads‐Protein A and incubating for 2 h at 4°C on a tube rotator. The sample was removed using a magnetic stand and transferred into a 1.5‐ml low adhesion tube. A 12.5‐µl sample was removed as the Input sample. A 50‐μl volume of GFP (A6455; Invitrogen, now ThermoFisher Scientific) coated Dynabeads was added to each sample and incubated for 4 h at 4°C.The samples were washed twice (5 min each at 4°C) with the following four cold‐wash buffers: low salt wash buffer, high salt wash buffer, 250 mm LiCl buffer, 0.5× Tris EDTA. Immunoprecipitated DNA was eluted from the Dynabeads by the addition of 50 µl of nuclei lysis buffer and a 30‐min incubation at 65°C on an Eppendorf ThermoMixer (Eppendorf, https://eppendorf.com). The elution was repeated a second time and the samples combined. Cross‐links were reversed by the addition of 6 µl of 5 m NaCl to the ChIP samples and an overnight incubation at 65°C. An 87.5‐µl volume of nuclei lysis buffer and 6 µl of 5 m NaCl was added to the input samples followed by overnight incubation at 65°C. The input and ChIP DNA was purified using a Qiagen PCR purification kit. Primers for ChIP‐qPCR are listed in Table S5. Fold enrichment was determined relative to a negative control, the transposon *TA3*.

Sequencing libraries were prepared from two biological replicates of input and ChIP DNA for stage‐6/7 flowers for both *AP1:AP1‐GR ap1 cal* and *AP1:AP1‐GR ap1 cal ANT:ANT‐VENUS ant‐4* using Accel‐NGS 2S DNA library kit (Swift Biosciences, https://swiftbiosci.com). The libraries were quantitated using the NEBNext Library Quant Kit for Illumina (New England Biolabs, https://international.neb.com) and sequenced on an Illumina HiSeq 2500 producing 150 base paired‐end reads. Sequence reads were aligned to the reference *A. thaliana* genome (version TAIR9, released June 2009) using bowtie 2. Examination of the coverage graphs revealed high reproducibility between the two ChIP‐Seq replicates. In addition, the input samples closely resembled the control untagged *AP1:AP1‐GR ap1 cal* samples. ANT binding peaks were identified using a visual analytics approach within the integrated genome browser (igb) (Freese *et al.*, 2016). Specifically, coverage graphs were generated for the combined data from the two replicates. A difference coverage graph was generated by subtracting coverage graphs of the untagged sample (*AP1:AP1‐GR ap1 cal*) from the coverage graphs for the tagged sample (*AP1:AP1‐GR ap1 cal ANT:ANT‐VENUS ant‐4*). Peaks were defined using the thresholding feature. A thresholding value of 5000 identified 90 peaks, whereas a thresholding value of 1000 identified 11 133 peaks. Further analyses were performed using the 1113 peaks identified with a threshold value of 1000. For each peak identified, chippeakanno was used to identify the gene with the closest transcription start site (TSS) (Zhu *et al.*, 2010). geneoverlap was used to compare gene lists for different ChIP‐Seq data sets. GO analyses were performed with amigo 2 (http://amigo.geneontology.org/amigo). Source code for bioinformatic analyses is available in the project ‘git’ repository https://bitbucket.org/krizeklab.

### Motif analysis with the MEME Suite

Genomic locations of putative ANT binding sites were determined using the fimo tool of meme suite (Grant *et al.*, 2011). The *in vitro* defined ANT binding motif was used as a position‐specific prior (Nole‐Wilson and Krizek, 2000). Putative ANT binding sites were identified using a *P* value of 0.001 or lower. Novel motif discovery was performed with meme‐chip.

### Petal measurements

Petal width, length and area were measured as described previously (Krizek, 2015).

## ACCESSION NUMBERS

RNA‐Seq sequences are available from the Sequence Read Archive (https://www.ncbi.nlm.nih.gov/sra) under accession number PRJNA539947. ChIP‐Seq sequences are available from the Sequence Read Archive (https://www.ncbi.nlm.nih.gov/sra) under accession number PRJNA593434. Version‐controlled source code used to process and analyze data is available from https://bitbucket.org/krizeklab. Sequence alignments and coverage graphs are available for interactive visualization within igb (Nicol *et al.*, 2009). To view the data in igb, readers may download and install the software from https://bioviz.org. Once installed, data sets from the study can be opened within igb by selecting the latest *A. thaliana* genome and then choosing RNA‐Seq and ChIP‐Seq folders within the Available Data Sets section of the Data Access Panel.

## AUTHOR CONTRIBUTIONS

BAK and AEL designed the research. BAK carried out the experiments. BAK, ICB, YYH, NF and AEL performed the data analyses. BAK wrote the article. All authors edited the article.

## CONFLICT OF INTEREST

The authors declare that they have no conflicts of interest.

## Supporting information


**Figure S2.** Interactive r shiny app tool to display gene expression data in control (C) and treated (T) *35S:ANT‐GR* samples. Control samples correspond to mock‐treated *35S:ANT‐GR* inflorescences whereas treated samples correspond to dex‐treated *35S:ANT‐GR* inflorescences. A. Sample RPKM for *AT1G48660* that shows gene expression values for each of four biological replicates. B. Group RPKM for *AT1G48660* which show average RPKM for the four replicates. C. Expression over time for *AT1G48660*. D. Gene info links for *AT1G48660*.
**Figure S3.** Hormone signaling pathways associated with changes in ANT activity. Blue indicates genes that are downregulated after induction of ANT activity. Orange indicates genes that are upregulated after induction of ANT activity. Mixed colored rectangles indicate classes in which some genes were downregulated whereas others were upregulated. The circles above and below the rectangle represent the number of downregulated (blue) and upregulated (orange) genes for such classes. Abbreviations: TFs, transcription factors.
**Figure S4.** ChIP‐qPCR confirms that ANT binds to genomic regions upstream or within genes associated with polarity specification (*KAN2, PHB*) and hormone signaling (*BEH4*, *RGA*). ChIP‐Seq coverage graphs for *KAN2* (A), *PHB* (B), *BEH4* (E), and *RGA* (G). Numbers below the gene indicate the regions tested for ANT binding by ChIP‐qPCR. ChIP‐qPCR data for *KAN2* (B), *PHB* (D), *BEH4* (F) and *RGA* (H). Grey bars show results from *AP1:AP1‐GR ap1 cal* and black bars show results from *AP1:AP1‐GR ap1 cal ANT:ANT‐VENUS ant*. Numbers on the *x*‐axis correspond to the genomic regions indicated in the ChIP‐Seq coverage graphs.
**Figure S5.** Secondary motif identified in some MEME‐1 sites. Sequence logos for the DAP‐Seq site of PLT1 (top), the MEME‐1 motif (middle) and the secondary motif identified in some MEME‐1 sites (bottom).
**Figure S6.** Pairwise comparison heat map displaying the Jaccard index degree of overlap among whole‐genome ChIP data sets of floral regulators and AIL/PLT transcription factors. The ANT data set shows the highest level of overlap with those of AP1, JAG and PLT2. This degree of overlap is less than that observed among the floral organ identity proteins AP3, PI and AG. The heat map was created with heatmapper (heatmapper.ca) (Sabicki *et al.*, 2016).
**Figure S7.** *spl8‐1* flowers are smaller than wild‐type flowers. A. Col flower (left) and *spl8‐1* flower (right). B. Graph showing petal width, length and area for Col and *spl8‐1* flowers. *P < 0.05 (Student's *t*‐test). Scale bar: 1 mm.
**Figure S8.** *AN3/GIF1* and *XTH9* expression within developing flowers overlaps with *ANT* expression. *AN3/GIF1* mRNA expression in the inflorescence meristem (A), stage‐2 flower (A), stage‐4 flower (B), stage‐6 flower (C), stage‐8 flower (D) and in the developing carpel (E). *XTH9* mRNA expression in the inflorescence meristem (F), stage‐2 flower (F), stage‐4 flower (G), stage‐7 flower (H), stage‐8 flower (I) and in the developing carpel (J). All pictures taken at the same magnification. Abbreviations: IM, inflorescence meristem; st 2, stage‐2 flowers; st 4, stage‐4 flowers; st 6, stage‐6 flowers; st 7, stage‐7 flowers; and st 8, stage‐8 flowers. Scale bar: 50 µm. All pictures were taken at the same magnification.

**Table S1.** Developmental genes differentially expressed after ANT‐GR activation.
**Table S2.** Hormone genes differentially expressed after ANT‐GR activation.
**Table S3.** Petal area, length and width in L*er*, *ant‐4* and *ANT:ANT‐VENUS ant‐4* flowers.
**Table S4.** Floral organ counts in L*er*, *ant‐4* and *ANT:ANT‐VENUS ant‐4* flowers at positions 1–30 on the inflorescence.
**Table S5.** Primers used in this study.

**Appendix S1.** Genes differentially expressed in *35S:ANT‐GR* inflorescences after dex treatment.

**Appendix S2.** Over‐represented gene ontology (GO) terms for *35S:ANT‐GR* DE genes.

**Appendix S3.** Genes associated with ANT ChIP‐Seq peaks.

**Appendix S4.** Over‐represented gene ontology (GO) terms for genes associated with ANT ChIP‐Seq peaks.

**Appendix S5.** Genes DE in *35S:ANT‐GR* and bound by ANT.

**Appendix S6.** Over‐represented gene ontology (GO) terms for genes DE in *35S:ANT‐GR* and bound by ANT.

Supplementary Material
