# Supplementary material for: The Arabidopsis transcription factor AINTEGUMENTA orchestrates patterning genes and auxin signaling in the establishment of floral growth and form
Source: Plant J. 2020 May 5;103(2):752–68. doi: 10.1111/tpj.14769 (PMC7369219; doi:10.1111/tpj.14769)
Supplement: Supplementary file 2 — Table S1. Developmental genes differentially expressed after ANT‐GR activation. Table S2. Hormone genes differentially expressed after ANT‐GR activation. Table S3. Petal area, length and width in Ler, ant‐4 and ANT:ANT‐VENUS ant‐4 flowers. Table S4. Floral organ counts in Ler, ant‐4 and ANT:ANT‐VENUS ant‐4 flowers at positions 1–30 on the inflorescence. Table S5. Primers used in this study. [file TPJ-103-752-s002.docx]

**Supplementary Tables**

**The Arabidopsis transcription factor AINTEGUMENTA orchestrates patterning genes and auxin signaling in the establishment of floral growth and form**

Beth A. Krizek^1*^, Ivory C. Blakley^2^, Yen-Yi Ho^3^, Nowlan Freese^2^, Ann E. Loraine^2^

^1^Department of Biological Sciences, University of South Carolina, Columbia, SC

^2^Department of Bioinformatics and Genomics, University of North Carolina at Charlotte, Charlotte, NC

^3^Department of Statistics, University of South Carolina, Columbia, SC

*Corresponding author

Tel: +1 803-777-1876

Email: [krizek@sc.edu](mailto:krizek@sc.edu)

**Table S1. Developmental genes differentially expressed after ANT-GR activation**

| **Process** | **Gene** | **Name** | **log2 fold change (hr)** |
| --- | --- | --- | --- |
| **Polarity specification** | AT1G69180 | *CRC* | -0.195 (4) |
|  | AT4G00180 | *YAB3* | 0.269 (2); 0.262 (4); 0.374 (8) |
|  | AT2G26580 | *YAB5* | 0.408 (2) |
|  | AT1G32240 | *KAN2* | 0.227 (4); 0.255 (8) |
|  | AT2G37630 | *AS1* | -0.235 (4); -0.175 (8) |
|  | AT2G34710 | *PHB* | 0.256 (4); 0.346 (8) |
|  | AT3G57130 | *BOP1* | -0.449 (4) |
|  |  |  |  |
| **Floral organ development** | AT1G69120 | *AP1* | -0.144 (4) |
|  | AT4G36920 | *AP2* | 0.22 (2) |
|  | AT1G24260 | *SEP3* | 0.175 (8) |
|  | AT1G02065 | *SPL8* | -0.341 (2); -0.589 (4); -0.534 (8) |
|  | AT5G07280 | *EMS1* | -0.14 (4) |
|  | AT4G36930 | *SPT* | 0.269 (2); 0.297 (4); 0.418 (8) |
|  | AT1G11130 | *SUB* | 0.154 (4); 0.165 (8) |
|  |  |  |  |
| **Meristem maintenance** | AT5G13290 | *CRN* | 0.383 (4); 0.43 (8) |
|  | AT4G20270 | *BAM3* | 0.182 (8) |
|  | AT5G45780 | *CIK4* | 0.269 (4) |
|  | AT3G28917 | *MIF2* | -0.441 (4) |
|  | AT4G02810 | *FAF1* | -0.746 (8) |
|  | AT1G03170 | *FAF2* | 0.409 (8) |
|  | AT5G19260 | *FAF3* | -0.48 (4) |

**Table S2. Hormone genes differentially expressed after ANT-GR activation**

| **Process** | **Gene** | **Name** | **log2 fold change (time point)** |
| --- | --- | --- | --- |
| **Cytokinin (CK)** |  |  |  |
| Synthesis/Metabolism | AT5G21482 | *CKX7* | 0.309 (2); 0.317 (8) |
| Transport | AT1G19770 | *PUP14* | 0.264 (4) |
| Signaling | AT2G01830 | *CRE1/AHK4* | -0.272 (4) |
| Response | AT1G10470 | *ARR4* | -0.407 (4); -0.243 (8) |
|  | AT1G19050 | *ARR7* | -0.541 (4); -0.477 (8) |
|  | AT1G80440 | *KMD1* | 0.886 (4) |
|  | AT1G15670 | *KMD2* | 0.362 (2) |
|  | AT3G59940 | *KMD4* | 0.437 (2); 0.931 (4); 0.435 (8) |
| **Auxin (IAA)** |  |  |  |
| Synthesis/Metabolism | AT1G70560 | *TAA1/WEI8* | 0.297 (4); 0.444 (8) |
|  | AT4G24670 | *TAR2* | -0.185 (4); -0.278 (8) |
|  | AT4G28720 | *YUC8* | 1.22 (8) |
|  | AT2G22330 | *CYP79B3* | -0.611 (8) |
|  | AT1G48660 | *GH3* family | 0.717 (2); 1.17 (4); 1.3 (8) |
|  | AT5G13350 | *GH3* family | -0.412 (2) |
|  | AT2G23170 | *GH3.3* | -0.231 (2) |
|  | AT5G54510 | *DFL1/GH3.6* | -0.159 (4) |
|  | AT2G47750 | *GH3.9* | -0.272 (4); -0.527 (8) |
|  | AT4G03400 | *DFL2/GH3-10* | -0.224 (2); -0.229 (4); -0.417 (8) |
| Transport | AT2G21050 | *LAX2* | 0.252 (8) |
|  | AT1G77690 | *LAX3* | -0.325 (2); -0.755 (4); -0.779 (8) |
|  | AT1G73590 | *PIN1* | 0.202 (4) |
|  | AT1G70940 | *PIN3* | -0.411 (2); -0.28 (4) |
|  | AT1G23080 | *PIN7* | -0.159 (4) |
|  | AT2G36910 | *ABCB1* | -0.177 (4) |
|  | AT2G47000 | *ABCB4* | 0.454 (8) |
|  | AT1G75500 | *WAT1* | -0.215 (4) |
| Signaling | AT3G62980 | *TIR1* | 0.144 (4) |
|  | AT3G26810 | *AFB2* | 0.185 (8) |
|  | AT1G12820 | *AFB3* | 0.172 (4) |
|  | AT1G30330 | *ARF6* | 0.132 (4) |
|  | AT2G46530 | *ARF11* | 0.376 (8) |
|  | AT3G61830 | *ARF18* | 0.198 (8) |
|  | AT1G04240 | *SHY2/IAA3* | 0.285 (8) |
|  | AT4G29080 | *PAP2/IAA27* | 0.14 (4) |
| Response | AT1G19840 | *SAUR53* | -0.423 (4) |
|  | AT5G18020 | *SAUR20* | -0.957 (4) |
|  | AT4G34760 | *SAUR50* | 0.346 (8) |
|  | AT4G38840 | *SAUR14* | -0.478 (8) |
| **Gibberellin (GA)** |  |  |  |
| Synthesis/Metabolism | AT1G15550 | *GA3OX1* | -0.54 (4) |
| Signaling | AT1G14920 | *GAI* | 0.133 (4); 0.181 (8) |
|  | AT2G01570 | *RGA* | 0.246 (2); 0.259 (4); 0.334 (8) |
|  | AT3G03450 | *RGL2* | -0.198 (4) |
| Response | AT1G75750 | *GASA1* | 0.209 (2) |
|  | AT1G74670 | *GASA6* | -0.235 (4) |
|  | AT5G14920 | *GASA14* | 0.475 (2); 0.33 (4); 0.335 (8) |
|  |  |  |  |
| **Abscisic Acid (ABA)** |  |  |  |
| Signaling | AT4G17870 | *PYR1* | 0.51 (2); 0.463 (4); 0.395 (8) |
|  | AT2G40330 | *PYL6* | 0.552 (2) |
|  | AT5G08590 | *SnRK2.1* | 0.134 (4); 0.196 (8) |
|  | AT5G63650 | *SnRK2.5* | 0.24 (8) |
| **Jasmonic Acid (JA)** |  |  |  |
| Synthesis/Metabolism | AT1G55020 | *LOX1* | 0.319 (2) |
|  | AT3G45140 | *LOX2* | -0.246 (4); -0.401 (8) |
|  | AT1G17420 | *LOX3* | -0.281 (8) |
|  | AT1G72520 | *LOX4* | 0.296 (4); -0.519 (8) |
|  | AT1G20510 | *OPCL1* | -0.363 (8) |
|  | AT5G42650 | *CYP74A* | -0.328 (8) |
|  | AT5G05600 | *JAO2* | -0.829 (8) |
|  | AT1G19640 | *JMT* | -0.334 (4); -0.334 (8) |
| Signaling | AT1G19180 | *JAZ1* | -0.338 (2); -0.245 (4); -0.489 (8) |
|  | AT1G74950 | *JAZ2* | -0.277 (8) |
|  | AT1G17380 | *JAZ5* | -0.361 (4); -0.756 (8) |
|  | AT1G72450 | *JAZ6* | -0.233 (4); -0.578 (8) |
|  | AT2G34600 | *JAZ7* | -1.07 (8) |
|  | AT1G70700 | *JAZ9* | -0.567 (8) |
|  | AT5G13220 | *JAZ10* | -0.219 (4); -0.438 (8) |
|  | AT3G22275 | *JAZ13* | 2.0 (8) |
|  | AT3G27810 | *MYB21* | -0.505 (8) |
|  | AT1G32640 | *MYC2* | -0.184 (8) |

**Table S3. Petal area, length and width in L*er*, *ant-4* and *ANT:ANT-VENUS ant-4* flowers**

|  | Petal area (mm^2^) | Petal length (mm) | Petal width (mm) |
| --- | --- | --- | --- |
| L*er* | 2.30 + 0.19 | 3.33 + 0.14 | 1.19 + 0.07 |
| *ant-4* | 1.17 + 0.13 | 2.79 + 0.18 | 0.76 +0.07 |
| *ANT:ANT-VENUS ant-4* | 2.37 + 0.20 | 3.47 + 0.16 | 1.15 + 0.07 |

**Table S4. Floral organ counts in L*er*, *ant-4* and *ANT:ANT-VENUS ant-4* flowers at positions 1-30 on the inflorescence**

|  | L*er* | *ant-4* | *ANT:ANT-VENUS ant-4* |
| --- | --- | --- | --- |
| **Whorl 1** |  |  |  |
| Se | 4.00 | 4.01 | 4.00 |
| Pe/Se |  | 0.01 |  |
|  |  |  |  |
| **Whorl 2** |  |  |  |
| Pe | 4.01 | 3.86 | 4.00 |
| filament |  | 0.01 |  |
|  |  |  |  |
| **Whorl 3** |  |  |  |
| St | 5.82 | 4.63 | 5.99 |
|  |  |  |  |
| **Whorl 4** |  |  |  |
| Ca | 2.00 | 2.00 | 2.00 |
|  |  |  |  |
| **Total of all whorls** | **15.83** | **14.52** | **15.99** |

**Table S5. Primers used in this study**

| **RT-qPCR primers** | | **Primer sequence (5' to 3')** |
| --- | --- | --- |
| *KAN2* | | KAN2-F: ACCTTGGCTCATGTTAAGTCC |
|  | | KAN2-R: AGATTGTCCTGACGAAGCTG |
| *PHB* | | PHB-F: TTTGCTCTGAGTTCCCCAAG |
|  | | PHB-R: CTGCTCGTAAGATACCATCCTTC |
| *BEH4* | | BEH4-F: TCTGTAACGAAGCTGGTTGG |
|  | | BEH4-R: CACCTATCTCCATACGCTCTAC |
| *RGA* | | RGA-F: ATGGATGTTTGATGTTGGGTTG |
|  | | RGA-R: GTCGAGAGTTTCCAAGCGG |
| *SPL8* | | SPL8-F: GCCGTAAATGTCACCAATCAG |
|  | | SPL8-R: GCTTTAACACCCGAATCGTTC |
| *AN3* | | AN3-RT1: CCAAGTGTGCATAGCCAGTA |
|  | | AN3-RT2: GTTGCTGTTGAGTCGCTTG |
| *XTH9* | | XTH9-F: TGGGCTATGGATCATTGTGTC |
|  | | XTH9-R: TGATTCAAACCCAGCTCCAG |
| *SRP2* | | SRP2-RT1: CCTTTCAAGCTTCTCCTTTTCG |
|  | | SRP2-RT2: CTTCACCAATGACGGAACATATG |
| **ChIP-qPCR primers** | |  |
| *KAN2* (fragment 1) | | KAN Pro1: AGGTGGAATGTAGACTAAGAAAAG |
|  | | KAN Pro2: CAATGACAAGGTAGTATGTAGTGAA |
| *KAN2* (fragment 2) | | KAN Pro5: CTCAATGATGAAATGGTTTTGGTC |
|  | | KAN Pro4: ACTGTTCGGACACAGAAGCATT |
| *PHB* (fragment 1) | | PHB Pro3: GCAGAGACTGTTTTGCGTG |
|  | | PHB Pro4: AATTACTACATAAGTTTCTTCTGGTTA |
| *PHB* (fragment 2) | | PHB Pro1: CACATTGAGTACAAATATGGACAC |
|  | | PHB Pro2: GTGTGGCGCGAGAAAGACT |
| *BEH4* (fragment 1) | | BEH4 Pro1: CATTAAACGACCAGTTTGAAC |
|  | | BEH4 Pro2: CATACATCGAGCACCATTACC |
| *BEH4* (fragment 2) | | BEH4 Pro3: GATGGAACTACTTACCGCAAG |
|  | | BEH4 Pro4: AGAATAGACTCGACCTGGAC |
| *RGA* (fragment 2) | | RGA Pro1: GACAAAAGTTTAATGATTCTCTTAGAC |
|  | | RGA Pro2: GAATGGTGGTGATGGGGC |
| *RGA* (fragment 1) | | RGA Pro3: AACTGAACCTGTAGGACTTAAAG |
|  | | RGA Pro4: GATGGAAGAACTGAAGATGCTC |
| *SPL8* (fragment 1) | | SPL8 Pro3: ACTCTCTCGTCTATGCTCAG |
|  | | SPL8 Pro4: AAAGAGAGAGAGATAAGAGGCA |
| *SPL8* (fragment 2) | | SPL8 Pro1: AGGCTCCATCAACAGTTGCT |
|  | | SPL8 Pro2: GCTCTCTCTCCCACCATC |
| *AN3* (fragment 1) | | AN3 Pro1: GATCAACACAATCAAACATCTACAAAG |
|  | | AN3 Pro2: GAGTTTTTAACCTAGTGGATTGAATTTG |
| *AN3* (fragment 2) | | AN3 Pro3: GGTCTCACGTCTCTATAATAAAGTATG |
|  | | AN3 Pro4: ATCTCGCTTTCTCTCCTCCAC |
| *AN3* (fragment 3) | | AN3 I1: GGTTCTGGAAGTTTCATTGATGGC |
|  | | AN3 I2: CTGAGATAAGGACCAGATGTTGTT |
| *XTH9* (fragment 1) | | XTH9-Pro1: CTGAAGAAGGAACTGAGGAATGG |
|  | | XTH9-Pro2: CTCATGATGAGATGACGCCAAATTA |
| *XTH9* (fragment 2) | | XTH9-Pro5: TTTGAGCTTCTGACACAAAAGGAGC |
|  | | XTH9-Pro4: CACTTTTGTACTACACAGAGGAC |
| *SRP2* (fragment 1) | | SRP2-Pro1: TTCCACAAGAAGCCCAATGCTC |
|  | | SRP2-Pro2: AAACACGGCTTTACCAGATTACCAG |
| *SRP2* (fragment 2) | | SRP2-Pro3: CATGTAAGGTTTGGAGGGAATATA |
|  | | SRP2-Pro4: GAGACGAGTGACCTCTAGGAC |
| **Cloning primers** | |  |
| *AN3* | AN3-1: CCTAGTCGACATGCAACAGCACCTGATGCAG | |
|  | AN3-2: CCTAGTCGACTCAATTCCCATCATCTGATGATTTC | |
| *XTH9* | XTH9-1: AGATTCTAGAATGGTCGGTATGGATTTGTTCAAATG | |
|  | XTH9-2: AGATTCTAGACTACAAATGACGATGATGTTGGCA | |

Data S1. Genes differentially expressed in *35S:ANT-GR* inflorescences after dex treatment

Data S2. Overrepresented Gene Ontology (GO) terms for *35S:ANT-GR* DE genes

Data S3. Genes associated with ANT ChIP-Seq peaks

Data S4. Overrepresented Gene Ontology (GO) terms for genes associated with ANT ChIP-Seq peaks

Data S5. Genes DE in *35S:ANT-GR* and bound by ANT

Data S6. Overrepresented Gene Ontology (GO) terms for genes DE in *35S:ANT-GR* and bound by ANT

**Methods S1.**

***In situ* hybridization**

Inflorescences were fixed, embedded, sectioned, hybridized and washed as described previously (Krizek *et al.* 2016). Templates for probe preparation for *AN3/GIF1* and *XTH9* were made by PCR amplification using the primers listed in Table S3, Phusion DNA polymerase and cDNA made from RNA isolated from Columbia inflorescences. The PCR products were cloned into the either the SalI (*AN3/GIF1*) or XbaI (*XTH9*) site of pGEM3z and sequenced. Antisense probes were made after linearization of AN3/pGEM3z with HindIII and XTH9/pGEM3Z with SalI and *in vitro* transcription with T7 RNA polymerase.
