# Supplementary material for: The Arabidopsis transcription factor AINTEGUMENTA orchestrates patterning genes and auxin signaling in the establishment of floral growth and form
Source: Plant J. 2020 May 5;103(2):752–68. doi: 10.1111/tpj.14769 (PMC7369219; doi:10.1111/tpj.14769)
Supplement: Supplementary file 9 — Supplementary Material [file TPJ-103-752-s009.docx]

**Supplementary Legends**

Figure S1. Dex treatment of *35S:ANT-GR* inflorescences results in larger flowers and male sterility. Mock (left) and dex (right) treated *35S:ANT-GR* flowers. Size bar is 1mm.

Figure S2. Interactive R Shiny app tool to display gene expression data in control (C) and treated (T) *35S:ANT-GR* samples. Control samples correspond to mock-treated *35S:ANT-GR* inflorescences while treated samples correspond to dex-treated *35S:ANT-GR* inflorescences. A. Sample RPKM for *AT1G48660* that shows gene expression values for each of four biological replicates. B. Group RPKM for *AT1G48660* which show average RPKM for the four replicates. C. Expression over time for *AT1G48660*. D. Gene info links for *AT1G48660*.

Figure S3. Hormone signaling pathways associated with changes in ANT activity. Blue indicates genes that are downregulated after induction of ANT activity. Orange indicates genes that are upregulated after induction of ANT activity. Mixed colored rectangles indicate classes in which some genes were downregulated while others were upregulated. The circles above and below the rectangle represent the number of downregulated (blue) and upregulated (orange) genes for such classes. Abbreviations: TFs, transcription factors.

Figure S4. ChIP-qPCR confirms that ANT binds to genomic regions upstream or within genes associated with polarity specification (*KAN2, PHB*) and hormone signaling (*BEH4*, *RGA*). ChIP-Seq coverage graphs for *KAN2* (A), *PHB* (B), *BEH4* (E), and *RGA* (G). Numbers below the gene indicate the regions tested for ANT binding by ChIP-qPCR. ChIP-qPCR data for *KAN2* (B), *PHB* (D), *BEH4* (F) and *RGA* (H). Grey bars show results from *AP1:AP1-GR ap1 cal* and black bars show results from *AP1:AP1-GR ap1 cal ANT:ANT-VENUS ant*. Numbers on the x axis correspond to the genomic regions indicated in the ChIP-Seq coverage graphs.

Figure S5. Secondary motif identified in some MEME-1 sites. Sequence logos for the DAP-Seq site of PLT1 (top), the MEME-1 motif (middle), and the secondary motif identified in some MEME-1 sites (bottom).

Figure S6. Pairwise comparison heat map displaying the Jaccard index degree of overlap among whole genome ChIP datasets of floral regulators and AIL/PLT transcription factors. The ANT data set shows the highest amount of overlap with those of AP1, JAG and PLT2. This degree of overlap is less than that observed among the floral organ identity proteins AP3, PI, and AG. The heat map was created with Heatmapper (heatmapper.ca) (Sabicki *et al.* 2016).

Figure S7. *spl8-1* flowers are smaller than wild-type flowers. A. Col flower (left) and *spl8-1* flower (right). B. Graph showing petal width, length and area for Col and *spl8-1* flowers. * indicates values with a *P* value less than 0.05 (Student's *t*-test). Size bar is 1mm.

Figure S8. *AN3/GIF1* and *XTH9* expression within developing flowers overlaps with *ANT* expression. *AN3/GIF1* mRNA expression in the inflorescence meristem (A), stage 2 flower (A), stage 4 flower (B), stage 6 flower (C), stage 8 flower (D), and in the developing carpel (E). *XTH9* mRNA expression in the inflorescence meristem (F), stage 2 flower (F), stage 4 flower (G), stage 7 flower (H), stage 8 flower (I), and in the developing carpel (J). All pictures taken at the same magnification. Abbreviations: IM (inflorescence meristem); st 2 (stage 2 flowers); st 4 (stage 4 flowers); st 6 (stage 6 flowers); st 7 (stage 7 flower); and st 8 (stage 8 flowers). Size bar is 50μm. All pictures were taken at the same magnification.

Table S1. Developmental genes differentially expressed after ANT-GR activation

Table S2. Hormone genes differentially expressed after ANT-GR activation

Table S3. Petal area, length and width in L*er*, *ant-4* and *ANT:ANT-VENUS ant-4* flowers

Table S4. Floral organ counts in L*er*, *ant-4* and *ANT:ANT-VENUS ant-4* flowers at positions 1-30 on the inflorescence

Table S5. Primers used in this study

Data S1. Genes differentially expressed in *35S:ANT-GR* inflorescences after dex treatment

Data S2. Overrepresented Gene Ontology (GO) terms for *35S:ANT-GR* DE genes

Data S3. Genes associated with ANT ChIP-Seq peaks
